# Abnormal Functional Connectivity of Posterior Cingulate Cortex Correlates With Phonemic Verbal Fluency Deficits in Major Depressive Disorder

**DOI:** 10.3389/fneur.2021.724874

**Published:** 2021-08-26

**Authors:** Danian Li, Hanyue Zhang, Yujie Liu, Xinyu Liang, Yaoping Chen, Yanting Zheng, Shijun Qiu, Ying Cui

**Affiliations:** ^1^Cerebropathy Center, The First Affiliated Hospital of Guangzhou University of Chinese Medicine, Guangzhou, China; ^2^Department of Radiology, The First Affiliated Hospital of Guangzhou University of Chinese Medicine, Guangzhou, China; ^3^Department of Radiology, Guangzhou First People's Hospital, School of Medicine, South China University of Technology, Guangzhou, China; ^4^Department of Radiology, The Third Affiliated Hospital of Sun Yat-sen University, Guangzhou, China; ^5^Cerebropathy Center, The Third Affiliated Hospital of Guangzhou Medical University, Guangzhou, China

**Keywords:** major depressive disorder, verbal fluency, fMRI, functional connectivity, posterior cingulate cortex

## Abstract

**Background:** Major depressive disorder (MDD) patients face an increased risk of developing cognitive impairments. One of the prominent cognitive impairments in MDD patients is verbal fluency deficit. Nonetheless, it is not clear which vulnerable brain region in MDD is interactively linked to verbal fluency deficit. It is important to gain an improved understanding for verbal fluency deficit in MDD.

**Methods:** Thirty-four MDD patients and 34 normal controls (NCs) completed resting-state fMRI (rs-fMRI) scan and a set of verbal fluency tests (semantic VFT and phonemic VFT). Fourteen brain regions from five brain networks/systems (central executive network, default mode network, salience network, limbic system, cerebellum) based on their vital role in MDD neuropathology were selected as seeds for functional connectivity (FC) analyses with the voxels in the whole brain. Finally, correlations between the z-score of the FCs from clusters showing significant between-group difference and z-score of the VFTs were calculated using Pearson correlation analyses.

**Results:** Increased FCs in MDD patients vs. NCs were identified between the bilateral posterior cingulate cortex (PCC) and the right inferior frontal gyrus (triangular part), in which the increased FC between the right PCC and the right inferior frontal gyrus (triangular part) was positively correlated with the z score of phonemic VFT in the MDD patients. Moreover, decreased FCs were identified between the right hippocampal gyrus and PCC, as well as left cerebellum Crus II and right parahippocampal gyrus in MDD patients vs. NCs.

**Conclusions:** The MDD patients have altered FCs among key brain regions in the default mode network, the central executive network, the limbic system, and the cerebellum. The increased FC between the right PCC and the right inferior frontal gyrus (triangular part) may be useful to better characterize pathophysiology of MDD and functional correlates of the phonemic verbal fluency deficit in MDD.

## Introduction

Major depressive disorder (MDD) is one of the most common and costly psychiatric disorders (1). In addition to emotion dysfunction (2), MDD patients face an increased risk of developing cognitive impairments (3). Among all the cognitive impairments, the deficit of verbal fluency, which requires a variety of executive function and non-executive function cognitive processes, has been found to be one of the prominent impairments in MDD (4). Interestingly, although only small improvements in cognitive impairments were found during the treatment of depression, verbal fluency was the most sensitive to improve in the treatment when compared to other cognitive domains (5). In light of these findings, it is important to gain a better understanding and find a potential biomarker for verbal fluency deficit in MDD, which could be of great clinical importance in terms of allowing early and accurate diagnosis (6).

Previous MDD studies often used verbal fluency test (VFT) in neuropsychological assessment to make the diagnosis of verbal fluency deficit (7). The VFT is a short test of verbal functioning (8). It typically consists of two tasks: semantic fluency (sometimes called category fluency) (9) and phonemic fluency (sometimes called letter fluency) (10). Two robust meta-analyses revealed the cognitive demands of the two tasks in verbal fluency (11, 12). They indicated that semantic verbal fluency was more related to semantic knowledge, semantic memory retrieval, language production, and strategy formation, while phonemic verbal fluency was more related to vocabulary, language production, memory retrieval, and strategy formation. In general patients with MDD, both semantic and phonemic verbal fluency measures could be detected to be impaired (4), and some studies have reported that semantic fluency is more impaired than phonemic fluency (13, 14), but others the reverse (15). The above results indicated a deficit of verbal fluency in MDD patients; however, the underlying brain functional alteration has not been fully revealed yet.

Resting-state functional MRI (rs-fMRI) has been widely used to investigate the neural mechanisms of brain dysfunctions (16) and to explore potential imaging biomarkers in various diseases (e.g., MDD, social anxiety disorder, and Alzheimer's disease) (17–19). By measuring fluctuations in blood-oxygen-level-dependent (BOLD) signals, rs-fMRI can be used to assess brain functional connectivity (FC). researchers have indicated that cognitive impairments in MDD are related to significant FC changes within and between several brain networks, such as the default mode network (DMN), central executive network (CEN), salience network (SN), and limbic system (LS) (20–23). Our previous study also showed that the crus II in the cerebellum was a promising biomarker for MDD diagnosis, and may be related to the cognitive impairments in MDD (24, 25). For both semantic and phonemic VFT, previous studies found patients with MDD had reduced activation in the ventrolateral prefrontal cortex, dorsolateral prefrontal cortex, and anterior cingulate cortex (26, 27), providing primary results for future work to reveal how brain functional alteration relates to verbal fluency deficit.

However, limitations exist in the previous studies. First, most MDD studies that evaluated the verbal fluency of patients by seed-based methods mostly chose seeds only from CEN. It is not clear which vulnerable brain region other than prefrontal regions in MDD is interactively linked to verbal fluency deficit, and whether this interaction is altered in MDD. Therefore, seeds from multiple MDD related networks would be more helpful in detecting the cognitive impairments. Second, the majority of the patients in the previous studies have a treatment history, but the resting-state networks are widely modulated by psychotropic medications (28). Therefore, a sample composed purely of first-episode and drug-naïve MDD patients may eliminate the possible confounding factors of medication use and achieve a more reliable result. In the current study, thirty-four first-episode and drug-naïve MDD patients and 34 normal controls (NCs) completed resting-state fMRI (rs-fMRI) scan and a set of VFTs. Fourteen brain regions from multiple MDD related brain networks/systems based on their vital role in MDD neuropathology were selected as seeds for FC analyses with the voxels in the whole brain. Finally, correlations between the z-score of the FCs from clusters showing significant between-group difference and z-score of the VFTs were calculated using Pearson correlation analyses. We hypothesized that MDD patients would show altered FC in the above brain regions, and some of the altered FC would be correlated with the VFT scores.

## Methods and Materials

### Participants

A total of 37 first-episode, treatment-naïve MDD patients and 38 NCs were included in this study. MDD patients were recruited from the psychological counseling outpatient clinic of the First Affiliated Hospital of Guangzhou University of Chinese Medicine from August 2019 to June 2020. The diagnosis of treatment-naïve, first-episode depression was made by two attending psychiatrists, each of whom had more than 10 years of experience in MDD diagnosis. The Diagnostic and Statistical Manual of Mental Disorders (DSM)-5 (29) and the Structured Clinical Interview for the DSM (SCID) was used to assess whether the diagnostic criteria were met (30). The 17-item Hamilton Depression Rating Scale (HDRS-17) (31) was also used to evaluate the severity of depression (32). Each patient self-reported a rough estimate of illness duration. The other inclusion criteria for MDD patients were as follows: (1) aged between 18 and 55 years old, (2) HDRS-17 score > 17, (3) right-handed native Chinese speaker, and (4) free of any history of neurological illness or any other psychiatric disorder according to the DSM-5. Exclusion criteria included (1) a history of any significant illness, (2) alcohol abuse [accessed by the Alcohol Use Disorders Identification Test (33)], and (3) contraindications to MRI scans. The NCs were all volunteers who were physically healthy based on their self-reported medical history and mentally healthy according to the Mini-International Neuropsychiatric Interview (MINI) (34) as applied by two psychologists. Besides, the HDRS-17 score of NCs was <7. This study was conducted in accordance with the Declaration of Helsinki. All participants provided written informed consent, and the study was approved by the Ethics Committee of the First Affiliated Hospital of Guangzhou University of Chinese Medicine, Guangzhou, China.

### Verbal Fluency Testing

According to the previous literature, VFTs were divided in two parts: semantic fluency task and phonemic fluency task (7). In the semantic fluency task, participants were asked to give as many Chinese words from a given category (animal) as possible in 1 min. They were instructed not to provide the same word twice, or words from the same family (e.g., “cat,” “kitty,” etc.). In the phonemic fluency task, participants were asked to generate as many distinct Chinese characters as possible that began with a specific initial consonant (Fa) within 1 min. The participants were instructed not to provide the same character twice. Short task instructions were provided orally by the researcher before the experiment. VFTs started with a 1-min semantic fluency task, followed by 0.5 min of rest, and ended with a phonemic fluency task. All the answers were reviewed by two trained psychometric technicians. The generated Chinese words were marked as either correct or incorrect responses based on the Modern Chinese Dictionary. Only the numbers of correct words were taken as a dependent variable in VFTs. Scores were obtained for both semantic and phonemic fluency tasks, separately.

Statistical analyses were performed using IBM SPSS Statistics version 23.0 (Chicago, IL, USA). Age and education level were compared using two-sample *t*-tests, gender was compared using a chi-squared test, and VFT (semantic VFT and phonemic VFT) scores between MDD patients and NCs were compared by using linear regression analyses (age, gender, and education level as covariates). Since previous literature (13, 14) has revealed a decreasing trend of VFT scores in MDD patients, we used a one-tailed two-sample *t*-test.

### Image Acquisition

All MRI data were acquired using a 3.0-T GE Signa HDxt scanner with an 8-channel head-coil within 3 days of diagnosis. The participants were instructed to close their eyes and refrain from thinking anything. Two radiologists made consensus decisions that all participants were free of visible brain abnormalities or any form of lesions based on thick-slice axial T1- and T2-weighted images as well as T2-weighted fluid-attenuated inversion recovery (T2-FLAIR) images. The parameters of rs-fMRI included TR/TE = 2,000/30 ms, flip angle = 90°, matrix size = 64 × 64, and slice spacing = 1.0 mm, FOV = 220 × 220 mm^2^, slice thickness = 3 mm, slice number = 36, scanning time = 6′10″ (185 volumes). The parameters of three-dimensional T1-weighted images (3D-T1WI) included slice thickness = 1 mm, no slice gap, matrix size = 256 × 256, field of view (FOV) = 256 × 256 mm^2^, TR/TE = 6.9/1.5 ms, inversion time = 450 ms, FA = 12°, and 188 slices.

### Image Pre-processing

Image preprocessing was performed using SPM12 (www.fil.ion.ucl.ac.uk/spm) and DPARSF version 2.3 (http://rfmri.org/DPARSF). The images were corrected for acquisition time intervals between slices and head motion between volumes. Data from 3 MDD patients and 4 NC were discarded because their maximum cumulative head motion exceeded 2 mm in translation or 2° in rotation along any direction, or the mean framewise displacement Jenkinson (FD_Jenkinson) exceeded 0.2 mm (35). Next, 3D-T1WI data were coregistered to the rs-fMRI data of the same subject and further segmented using unified segment (http://www.fil.ion.ucl.ac.uk/spm) and registered to the standard Montreal Neurological Institutes (MNI) space using diffeomorphic anatomical registration through exponentiated Lie algebra (DARTEL). The rs-fMRI data were then warped to MNI space according to the generated deformation field and smoothed with a Gaussian kernel of 6 mm full width at half maximum (FWHM). Several nuisance signals, including the Friston-24 head motion parameters and mean signals from cerebrospinal fluid and white matter, were regressed out from the rs-fMRI data. Then, linear detrending and bandpass filtering (0.01–0.08 Hz) were performed to reduce low-frequency drift and high-frequency noise.

### FC Analysis

We specified 14 ROIs from AAL atlas (bilateral dorsolateral prefrontal cortex, bilateral insula, bilateral PCC, bilateral hippocampal gyrus, bilateral amygdala, bilateral thalamus, and bilateral Crus II in the cerebellum) from DMN, CEN, SN, LS, and cerebellum based on their vital role in MDD neuropathology. Using DPARSF version 2.3 (http://rfmri.org/DPARSF), we computed Pearson correlation coefficients between the mean time series of each ROI and that of each voxel of the whole brain. Then, a Fisher *r*-to-*z* transformation was used to convert the correlation coefficient to *z* values to improve normality. Finally, we obtained z-score of the FC maps of each individual for further analysis. Next, we used SPM 12 (www.fil.ion.ucl.ac.uk/spm) to perform two-sample *t*-tests (gender, age, and education as covariates) to determine areas with significantly different FCs to the ROIs between MDD patients and NCs. We used *P* < 0.001 for the cluster-forming threshold and implemented a family-wise error (FWE) correction approach at the cluster level. All results survived whole-brain cluster correction (*P*_FWE_ < 0.05).

### Correlation Between FC and VFT Scores

First, to identify the confounders influencing performance of VFT scores, a linear multiple regression analysis was performed for each dependent variable with age, gender, and education as predictors. Age and education were entered in the analyses as continuous variables, while gender was coded 1 for men and 2 for women. Interactions between predictors were tested. None of the interactions were significant so they were not retained in the final models. All statistical analyses were performed using SPSS software (version 23.0) with the alpha level set at 0.05. The residual was treated as the z-score of VFT. Then the correlations between the mean z-score of the FCs from clusters showing the significant between-group difference and z-score of VFTs (semantic VFT score and phonemic VFT score) were calculated using Pearson correlation analyses. *P* < 0.05 after Bonferroni correction {i.e., *P*_*uncorrected*_/[2 (semantic VFT and phonemic VFT) ^*^ 4 (number of significant different between-group FCs)]} was considered significant.

## Results

### Demographic and Clinical Characteristics

A total of 34 MDD patients (25 females, 9 males; mean age: 29.41 years) and 34 NCs (24 females, 10 males; mean age: 30.09 years) with fMRI data and VFT scores were included in the correlation analyses. No significant difference was found between the 34 MDD patients and the 34 NCs in terms of age, gender, education level, and the MDD patients had significantly lower semantic VFT and phonemic VFT scores than the NCs (*P*_*corrected*_ < 0.05). See details in Table 1.

**Table 1:** Demographic characteristics and VFT performance of the participants.

**Characteristics**	**MDD (*n* = 34)**	**NC (*n* = 34)**	***t*/χ^**2**^**	***P-*value**
Age, years	29.41 ± 8.27^§^	30.09 ± 10.88^§^	−0.29	0.77^†^
Gender (F/M)	25/9	24/10	0.07	0.79^‡^
Education (yrs.)	13.00 ± 3.44^§^	13.68 ± 3.07^§^	−0.86	0.40^†^
Illness duration (mo.)	7.81 ± 8.46^§^	NA	NA	NA
HDRS-17	21.85 ± 2.25^§^	NA	NA	NA
Semantic VFT score	18.15 ± 5.77^§^	21.47 ± 4.82^§^	2.44	0.01^¶^
Phonemic VFT score	8.15 ± 4.34^§^	9.91 ± 3.98^§^	1.86	0.03^¶^

*MDD, major depressive disorder; NC, normal control; HDRS-17, 17-item Hamilton Depression Rating Scale; VFT, verbal fluency test*.

§ *Mean ± standard deviation (SD)*.

† *The P-values were obtained by two-sample t-tests*.

‡ *The P-value was obtained by a chi-squared test*.

¶ *The P-values were obtained by linear regression analyses. Age, gender, and education level were included as covariates*.

### MDD-Related FC Alterations

All participants were free of any visible brain abnormality or any form of lesion based on thick-slice axial T1- and T2-weighted images as well as T2-FLAIR images.

**Figure 1:**
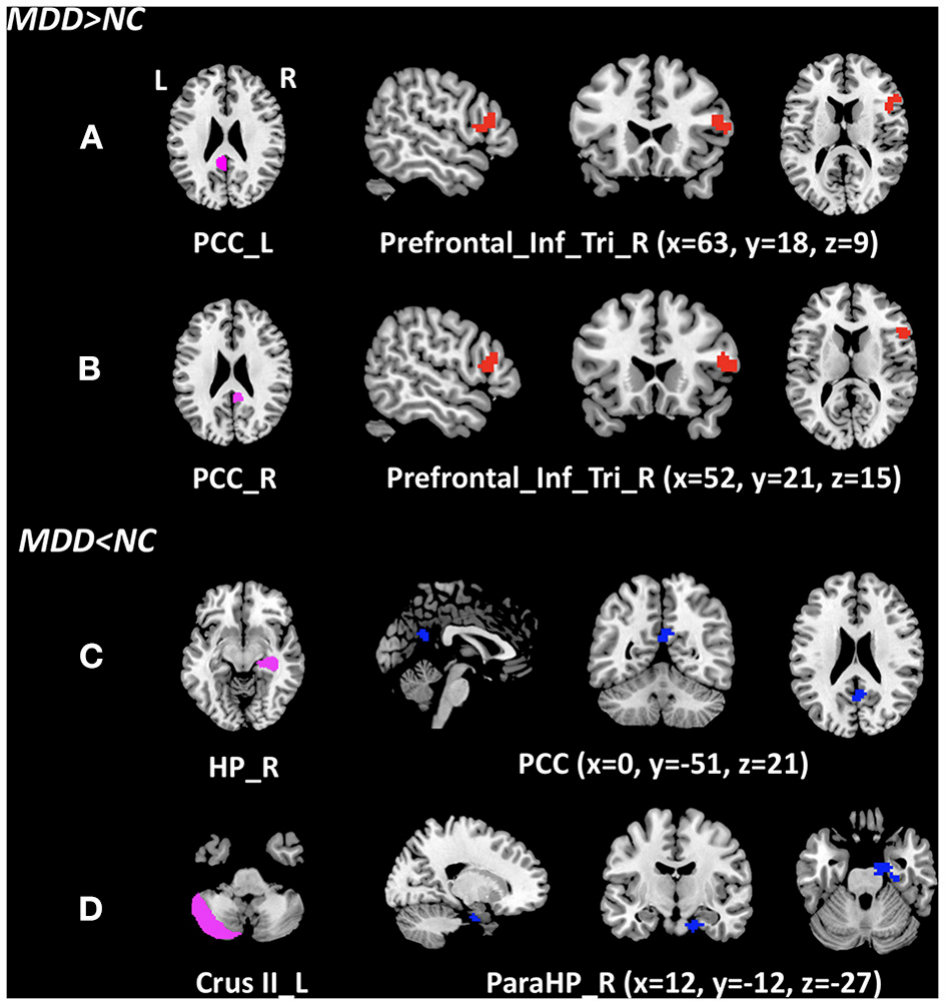
Clusters of between-group differences of z-score of the FC with age, gender, education level, and center adjusted (*P* < 0.05, FWE corrected). Compared to the NCs, significantly increased z-score of the FCs in MDD patients were found between **(A)** the left posterior cingulate cortex and the right inferior frontal gyrus (triangular part); **(B)** the right posterior cingulate cortex and the right inferior frontal gyrus (triangular part); significantly decreased z-score of the FCs in MDD patients were found **(C)** the right hippocampal gyrus and the PCC; and **(D)** left cerebellum Crus II and right parahippocampal gyrus. MDD, major depressive disorder; NC, normal control; cluster in red indicates the increased FC with the ROI, while blue indicates the decreased FC; PCC_L, left posterior cingulate cortex; PCC_R, right posterior cingulate cortex; Prefrontal_Inf_Tri_R, right inferior frontal gyrus (triangular part); HP_R, right hippocampal gyrus; Crus II_L, left Crus II in the cerebellum; PareHP_R, right parahippocampal gyrus; x, y, z, Montreal Neurological Institutes coordinates; L, left; R, right.

Significant differences were found in the z-score of the FC of four ROIs between MDD and NCs. As shown in Table 2 and Figure 1. Increased z-score of the FCs in MDD patients vs. NCs were identified between bilateral posterior cingulate cortex (PCC) and the right inferior frontal gyrus (triangular part). Decreased z-score of the FCs were identified between the right hippocampal gyrus and PCC, as well as left cerebellum Crus II and right parahippocampal gyrus in MDD patients vs. NCs.

**Table 2:** MDD-related FC alterations.

**Cluster**	**Seed**	**Area**	**Cluster size**	**Peak MNI coordinates**			**Peak T**
				**x**	**y**	**z**	
**MDD > NC**							
1	PCC_L	Frontal_Inf_Tri_R	68	63	18	9	4.29
2	PCC_R	Frontal_Inf_Tri_R	55	51	21	15	4.51
**MDD < NC**							
3	HP_R	PCC_L	40	0	−51	21	3.97
4	Crus II_L	ParaHP_R	45	12	−12	−27	4.99

*MDD, major depressive disorder; NC, normal control; PCC_L, left posterior cingulate cortex; PCC_R, right posterior cingulate cortex; Prefrontal_Inf_Tri_R, right inferior frontal gyrus (triangular part); HP_R, right hippocampal gyrus; Crus II_L, left Crus II in the cerebellum; PareHP_R, right parahippocampal gyrus; x, y, z, Montreal Neurological Institutes coordinates*.

### Correlations Between Altered FC and VFT Scores

**Figure 2:**
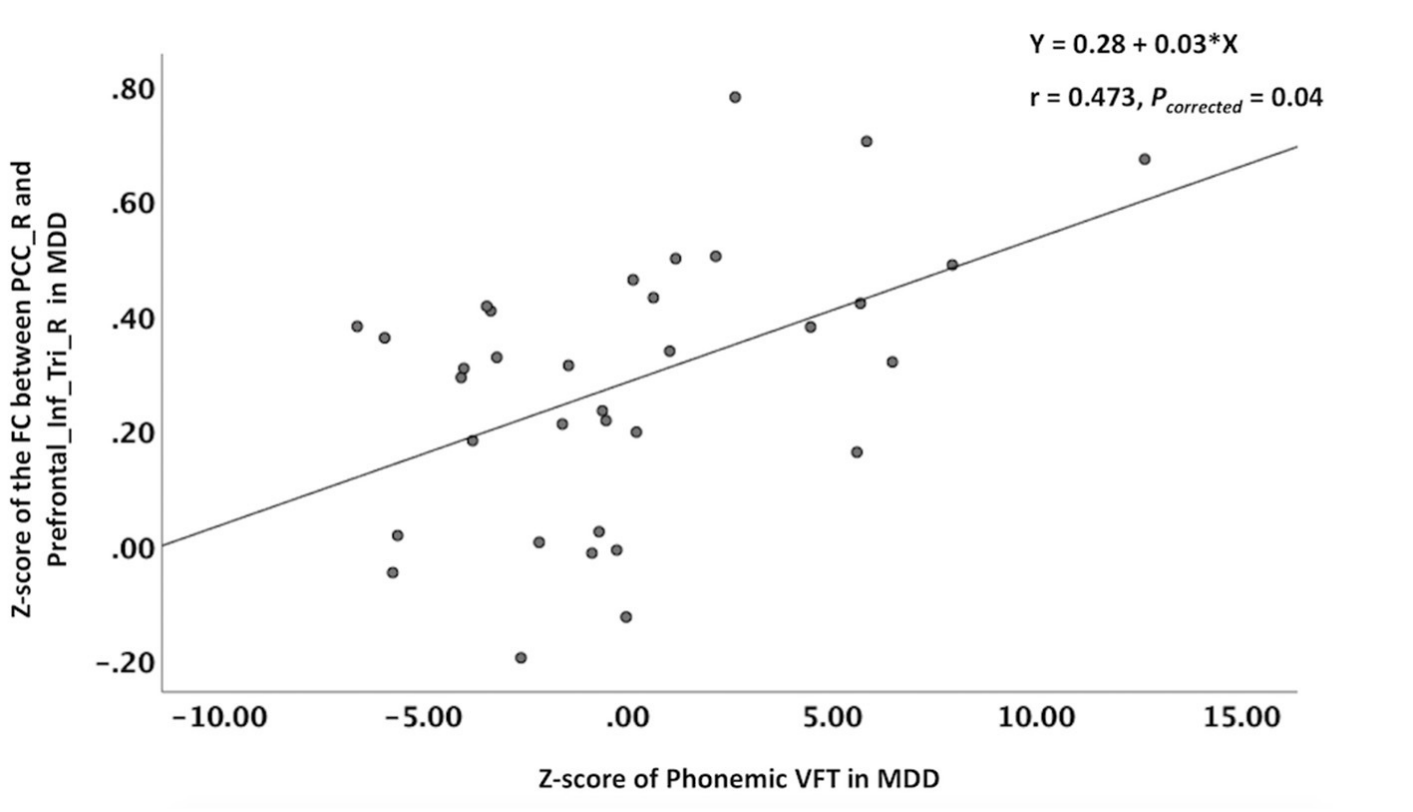
Correlations between altered z-score of the FC and the z-score of the VFT. The phonemic VFT score was positively correlated with the z-score of the FC between the right PCC and the right inferior frontal gyrus (triangular part). VFT, verbal fluency test; PCC_R, right posterior cingulate cortex; Prefrontal_Inf_Tri_R, right inferior frontal gyrus (triangular part).

In the MDD patients, the z-score of the phonemic VFT score was positively correlated with the z-score of the FC between the right PCC and the right inferior frontal gyrus (triangular part) (*r* = 0.473, *P*_corrected_ = 0.04), and the equations was *Y* = 0.28 + 0.03^*^X (Figure 2). Although the MDD patients had significantly lower semantic VFT score than the NCs, no correlation was found between any z-score of the FC and z-score of the semantic VFT in the MDD patients or the NCs.

## Discussion

In this study, we analyzed the FC differences of 14 AAL brain regions from multiple networks/systems (DMN, CEN, SN, LS and cerebellum) with the voxels of the whole brain, and correlated the altered FCs with VFT scores between 34 first-episode, drug-naïve MDD patients and 34 NCs. The result showed that significant FC differences between groups were identified among the brain regions and clusters in the DMN, CEN, LS, and cerebellum. Besides, the MDD patients performed worse in both semantic and phonemic VFT, and the z-score of the phonemic VFT was correlated with the z-score of the FC between the right PCC and the right inferior frontal gyrus. Our findings offer a novel insight into the pathophysiological mechanisms of verbal fluency deficit in MDD.

### MDD-Related FC Alterations

Studies over the past two decades have shown that the DMN, CEN, SN, LS and cerebellum support emotion regulation and higher cognitive functions in MDD (36). In this study, we observed several discriminative brain regions contributing to MDD-related FC alterations, including the bilateral PCC in the DMN, the right inferior frontal gyrus (triangular part) in the CEN, the right hippocampal gyrus and the right parahippocampal gyrus in the LS, as well as the left crus II in the cerebellum. As we know, the PCC is involved in memory formation, sensory monitoring and stereotypes, and plays a pivotal role in the DMN, which provides the neural substrate for depressive rumination (37). The increased FCs between the bilateral PCC and the right inferior frontal gyrus (triangular part) in MDD confirmed here may lead emotion related language processing problem, for the inferior frontal gyrus (triangular part) contributes to propositional language comprehension, as in the dominant cortical hemisphere it contributes to the Broca's area (38). This result is in accordance with the previous study of Rolls et al. (39).

Multiple MDD studies have focused on typically impaired brain networks such as DMN and LS because of their important roles in emotion processing and antidepressant action (23). Our results also indicated that the FC between the right hippocampal gyrus and PCC has been altered in the first-episode and drug-naïve MDD patients. The hippocampus in the limbic system is believed to mediate emotion regulation and memory processing. It mediates episodic memory, stress and negative emotion (40). The current finding of the hippocampal gyrus is consistent with the previous study (41), indicating a possible relationship with worse emotion regulation and poor episodic memory ability, for the PCC provides a route into the hippocampal memory system (42, 43), and is implicated in episodic memory including autobiographical memory (44, 45).

The cerebellum has been considered for a long time to play a role solely in motor coordination. However, recent studies have shown that the cerebellum also plays a key role in many motors, cognitive, and emotional processes (46). Besides, according to a new research, the Crus II in the cerebellum is specialized for social mentalizing and emotional self-experiences (47). In our previous study, we have reported the importance of the crus II in the cerebellum as a promising biomarker for MDD diagnosis (24, 25). Another study of geriatric depression also showed significantly reduced FC between the crus II and the ventromedial prefrontal cortex (48). In this study, we observed a decreased FC between the left crus II and right parahippocampal gyrus. Since the cerebellum is believed to be coupled with cerebral association areas (e.g., DMN), and the parahippocampal gyrus is part of the DMN that plays an important role in memory encoding and retrieval (49), the decreased FC confirmed here may suggest poor memory based on worse emotional self-experiences in MDD patients. We speculate such altered FC may provide the first evidence that the left crus II coupled with subcortical areas in the development of MDD.

### Correlations Between Altered FC and Clinical Scores

The prominent finding in this study was both semantic and phonemic verbal fluency deficit in MDD patients, indicating that the semantic knowledge, memory retrieval, vocabulary, language production, and strategy formation may be impaired in patients with MDD (4). This result was in accordance with some of the previous studies. Other studies have reported the semantic fluency is more impaired than phonemic fluency (13, 14). The possible reason is that semantic fluency may place heavier demands on switching, and particularly on selecting what to switch to, since category cues are likely to lead to the activation of many category members, which then compete for production (50). This explanation demonstrates impairments on shifting tasks in MDD that might lead to switching deficits in verbal fluency tasks.

Previous studies have found patients with MDD had reduced activation in ventrolateral prefrontal cortex, dorsolateral prefrontal cortex, and anterior cingulate cortex (26, 27), providing primary results for our work to reveal how brain functional alteration relates to verbal fluency deficit. In this study, we found that the z-score of the phonemic VFT was positively correlated with the z-score of the FC between the right PCC and the right inferior frontal gyrus (triangular part). As mentioned in the method, in the VFT, the participants were asked to give as many Chinese words from a given category (semantic VFT) or began with a specific initial consonant (phonemic VFT) as possible in a certain time. They were instructed not to provide the same word twice, or words from the same family. This top-down retrieval depends on conscious control. In order to home in on the desired information, some selection must occur. This selection is thought to occur post-retrieval in the mid-ventral lateral prefrontal cortex), which corresponds generally to the location of the triangular part (51). We suggest that the increased FC between the right PCC and the right inferior frontal gyrus (triangular part) may reverse the impacts on verbal fluency by pathological conditions of MDD. In other words, increased FC between PCC and frontal cortex seem to support a preserved verbal fluency ability in the MDD patients.

Although the MDD patients had significantly lower semantic VFT score than the NCs, no correlation was found between any z-score of the FC and z-score of the semantic VFT in the MDD patients or the NCs. However, there is a theory that the triangular part is especially involved in the semantic processing of language, as opposed to phonological processing. That is, the triangular part is thought to be more involved in deciphering the meaning of words rather than trying to decide what the word is based on the sound that goes into the ear (52). The inconsistency of the above results highlighted the importance of replicating the previous studies with a larger sample size of MDD patients.

### Limitations

There are several limitations to the study. First, the sample size of the patient with MDD is relatively small. Therefore, a larger sample size is needed in our future work. Besides, we only recruited only first-episode, drug-naïve MDD patients. Selecting this group of MDD patients eliminates possible confounding factors such as illness duration and medication use (28). However, different MDD subtypes could have different neurobiological mechanisms and should be investigated separately in the future (53). Third, we used only one imaging modality, but other modalities also provide valuable diagnostic information and could be used jointly with our protocol to improve diagnosis. Finally, we only use verbal fluency test in this study, but other cognitive tests, such as language-specific tasks may reveal more aspects of cognitive impairments in MDD. Further studies that include more cognitive tasks will be helpful to interpret this issue.

## Conclusions

The MDD patients have altered FCs among key brain regions in the default mode network, the central executive network, the limbic system, and the cerebellum. The increased FC between the right PCC and the right inferior frontal gyrus (triangular part) may be useful to better characterize pathophysiology of MDD and functional correlates of the phonemic verbal fluency deficit in MDD.

## Data Availability Statement

The raw data supporting the conclusions of this article will be made available by the authors, without undue reservation.

## Ethics Statement

The studies involving human participants were reviewed and approved by the First Affiliated Hospital of Guangzhou University of Chinese Medicine, Guangzhou, China. The patients/participants provided their written informed consent to participate in this study. Written informed consent was obtained from the individual(s) for the publication of any potentially identifiable images or data included in this article.

## Author Contributions

DL, HZ, SQ, and YCu contributed to conception and design of the study. DL, HZ, YL, XL, YCh, and YZ organized the data. DL performed the data analysis and drafted the manuscript. All authors revised the manuscript and read and approved the submitted version.

## Conflict of Interest

The authors declare that the research was conducted in the absence of any commercial or financial relationships that could be construed as a potential conflict of interest.

## Publisher's Note

All claims expressed in this article are solely those of the authors and do not necessarily represent those of their affiliated organizations, or those of the publisher, the editors and the reviewers. Any product that may be evaluated in this article, or claim that may be made by its manufacturer, is not guaranteed or endorsed by the publisher.
